# Application of the In Vitro HoxB8 Model System to Characterize the Contributions of Neutrophil–LPS Interaction to Periodontal Disease

**DOI:** 10.3390/pathogens9070530

**Published:** 2020-07-01

**Authors:** Maja Sochalska, Magdalena B Stańczyk, Maria Użarowska, Natalia Zubrzycka, Susanne Kirschnek, Aleksander M Grabiec, Tomasz Kantyka, Jan Potempa

**Affiliations:** 1 Department of Microbiology, Faculty of Biochemistry, Biophysics and Biotechnology, Jagiellonian University, 30-387 Krakow, Poland; magdabasiastanczyk@gmail.com (M.B.S.); maria.uzarowska@gmail.com (M.U.); natalia.zubrzycka@doctoral.uj.edu.pl (N.Z.); aleksander.grabiec@uj.edu.pl (A.M.G.); jan.potempa@uj.edu.pl (J.P.); 2 Institute of Medical Microbiology and Hygiene, Medical Center–University of Freiburg, Faculty of Medicine, 79104 Freiburg, Germany; susanne.kirschnek@uniklinik-freiburg.de; 3 Malopolska Centre of Biotechnology, Jagiellonian University, 30-387 Krakow, Poland; tomasz.kantyka@uj.edu.pl; 4 Broegelmann Research Laboratory, Department of Clinical Science, University of Bergen, 5020 Bergen, Norway; 5 Department of Oral Immunity and Infectious Diseases, University of Louisville, School of Dentistry, Louisville, KY 40202, USA

**Keywords:** *Porphyromonas gingivalis*, innate immune system, virulence factors, apoptosis, neutrophil biology

## Abstract

(1) Background: Studying neutrophils in vitro is difficult since these cells are terminally differentiated and are easily activated during isolation. At the same time, most of the available model cell lines are associated with certain limitations, such as functional deficiency or a lack of expression of surface markers characteristic of neutrophils. *P. gingivalis* is a periodontopathogen that causes dysbiosis in subgingival bacterial biofilm. This triggers the accumulation of functional neutrophils in the periodontium. However, until now, the specific effects of *P. gingivalis*-derived lipopolysaccharide on neutrophil functions have not been analyzed. (2) Methods: The impact of two variants of commercially available *P. gingivalis* endotoxin on neutrophil functions was tested using the HoxB8 in vitro system that is well suited to analyze neutrophil response to different stimuli in a controlled manner. (3) Results: The Standard *P. gingivalis* lipopolysaccharide (LPS), known to activate cells through Toll-like receptor 2 (TLR2)- and Toll-like receptor 4 (TLR4)-dependent pathways, prolonged neutrophil survival and exhibited pro-inflammatory effects. In contrast, Ultrapure LPS, binding exclusively to TLR4, neither protected neutrophils from apoptosis, nor induced an inflammatory response. (4) Conclusion: Two variants of *P. gingivalis*-derived LPS elicited effects on neutrophils and, based on the obtained results, we concluded that the engagement of both TLR2 and TLR4 is required for the manipulation of survival and the stimulation of immune responses of HoxB8 neutrophils.

## 1. Introduction

Studying neutrophils, also called polymorphonuclear leukocytes (PMNs), in vitro is difficult since these cells are easily activated during isolation, die rapidly in culture and only a limited amount of neutrophils can be isolated from mice. By now, many in vitro myeloid models devoted to study the process of neutrophil differentiation and functionality have been established. Those include factor-dependent immortalized human myeloid cell lines, i.e., promyelocytic NB-4 and myeloblastic HL-60 human leukemic cell lines that undergo differentiation in the presence of retinoic acid [1]. However, it was reported that these cell lines fail to terminally differentiate into mature neutrophils, as they are lacking some secondary granule proteins that are expressed at the late stages of the neutrophil maturation process [2].

Alternatively, many murine-inducible model cell lines are available that express the full range of neutrophil maturation markers. These include (i) Stem Cell Factor (SCF)- and Granulocyte-Macrophage Colony-Stimulating Factor (GM-CSF)-dependent lymphohematopoietic progenitor cell line EML/EPRO (erythroid myeloid lymphoid/early promyelocytic), which undergoes cytokine or retinoic acid maturation [3] or (ii) the 32D clone3 myeloblastic cell line, which differentiates in the presence of GM-CSF [4]. While differentiated 32Dcl3 cells are functionally similar to ex vivo isolated mouse neutrophils, they fail to produce superoxides and many of these cells undergo apoptosis already during the differentiation process [4]. The GM-CSF-dependent neutrophil progenitor EML/EPRO cell line is another option. These cells are capable of differentiating into neutrophils through a two-step process, which is long, as it takes 10 days. Another drawback of this model is the loss of a significant number of cells, which die during the selection process. Furthermore, Gr-1 expression was reported not to be significantly upregulated during EPRO cells maturation into neutrophils [3]. All these problems were solved by applying a conditional estrogen-inducible HoxB8 overexpression system to immortalize murine bone marrow neutrophil progenitors, which can be maturated into fully functional cells, indistinguishable from differentiated mouse bone marrow neutrophils [5,6].

It is a well-documented innate immunity paradigm that LPS is recognized by Toll-like receptor 4 (TLR4) of the host innate immune system, which induces pro-inflammatory cytokine production and triggers bacteria elimination [7]. However, as binding of LPS to TLR4 is true for most of the Gram-negative species, the *Porphyromonas gingivalis*-derived LPS is also a potent activator of TLR2 [8]. *P. gingivalis* is an anaerobic bacterium classified as a keystone pathogen that can cause deregulated inflammation and periodontitis in hosts without any apparent genetic predispositions [9]. 

However, until now, the specific effects of *P. gingivalis*-derived lipopolysaccharides on neutrophil survival and functions remained unraveled. Moreover, we aimed to establish a high-throughput platform for the identification of new therapeutic targets for the treatment of periodontitis by utilizing the HoxB8 in vitro model. By employing the HoxB8 system, we showed that the engagement of both TLR2 and TLR4 is required for the manipulation of neutrophil survival since Standard lipopolysaccharide, but not Ultrapure LPS from periodontal pathogen *P. gingivalis*, protects neutrophils from apoptosis. The process was accompanied by the enhanced production of Tumour Necrosis Factor-alpha (TNF-α) and reactive oxygen species (ROS) and was dependent on *P. gingivalis* LPS recognition, since endotoxin inactivation by polymyxin B significantly reduced neutrophil survival and pro-inflammatory responses. These findings were confirmed using primary human neutrophils. 

## 2. Results

### 2.1. A New Perspective in Periodontitis Research: The HoxB8 System

**Figure 1:**
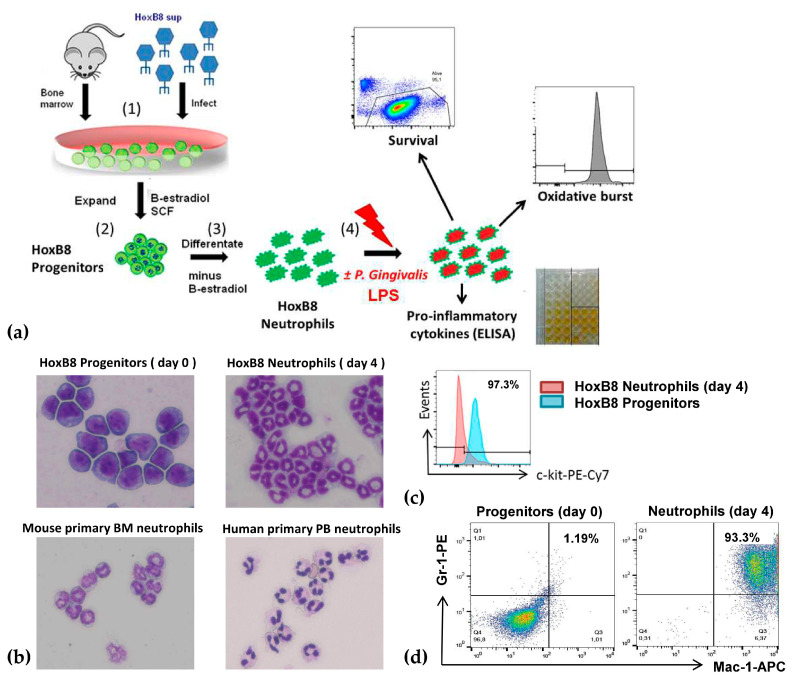
A novel approach to analyze neutrophil contribution to periodontitis. (**a**) Experimental workflow. (1) Bone marrow cells isolated from mice were infected with retrovirus encoding estrogen-dependent HoxB8. (2) Progenitors were cultured with β-estradiol and Stem Cell Factor (SCF). (3) β-estradiol withdrawal led to polymorphonuclear leukocyte (PMN) differentiation after 4 days. (4) PMNs were incubated with or without *P. gingivalis*-derived LPS to determine pathogen-mediated or basal survival and function of neutrophils, such as the release of pro-inflammatory cytokines or ROS generation. (**b**) Representative picture of Giemsa stainings: HoxB8 progenitors and neutrophils in culture on day 4 of differentiation (upper pictures) compared with primary bone marrow (BM) murine and peripheral blood (PB) human neutrophils (lower pictures); the multi-lobed nucleus characteristic of PMNs is visible in HoxB8 and primary neutrophils. (**c**) HoxB8 progenitor cells (blue line) during differentiation in vitro on day 4 downregulate the expression of surface markers characteristic of progenitors (red line), such as c-kit (CD117), while they (**d**) upregulate the expression of neutrophil markers, i.e., Gr-1 (Ly6G) and Mac-1 (CD11b) as analyzed by flow cytometry and depicted by histogram overlays (**c**) and dot plots (**d**).

HoxB8 neutrophil progenitor lines were generated from bone marrow cells by transduction with the conditional HoxB8-ER fusion protein, as described before [5]. Cells were cultured in a medium containing estrogen in order to maintain the progenitor-like phenotype (Figure 1b, upper left panel) characterized by a high expression of Sca-1 or c-kit (CD117; Figure 1c, blue line) and a low expression of Gr-1 (Ly6G) and Mac-1 (CD11b) surface markers (Figure 1d, left panel). SCF was added to the cell culture medium to ensure the survival and proliferation of neutrophil progenitors (Figure 1a: (1) and (2)). Upon the withdrawal of estrogen from cultures, HoxB8 expression was terminated and progenitors started to differentiate (Figure 1a: (3)). The expression of Sca-1 and c-kit (Figure 1c, red line) was downregulated while the expression of specific PMN surface markers, Gr-1 and Mac-1 (Figure 1d, right panel) was upregulated as verified by flow cytometry (Figure 1c,d). Moreover, on day four of the differentiation process, Giemsa staining documented the presence of a multi-lobed nucleus, the neutrophil hallmark (Figure 1b, upper right panel). In this way, HoxB8 PMNs were not only morphologically equivalent to ex vivo isolated primary murine and human neutrophils (Figure 1b, lower panels), but also functionally equivalent. The latter was documented by HoxB8 PMNs’ ability to induce phagocytosis and oxidative burst (Figure 1a (4)), as well as pro-inflammatory cytokine release (i.e., mTNF-α) and production of reactive oxygen species (Figure 1a (4)).

### 2.2. Two Variants of LPSs Isolated from P. gingivalis Differentially Impact Neutrophil Survival

Different *P. gingivalis* endotoxins were described to bind not only TLR4, but also TLR2 [8]. Therefore, we have decided to analyze and directly compare the impact of two *P. gingivalis* lipopolysaccharides on neutrophil survival and functions. Two variants of LPS isolated from *P. gingivalis* are commercially available [10], one activating only TLR4 signaling (so-called Ultrapure) and the other utilizing not only TLR4, but also TLR2 signaling pathways (so-called Standard) [8].

**Figure 2:**
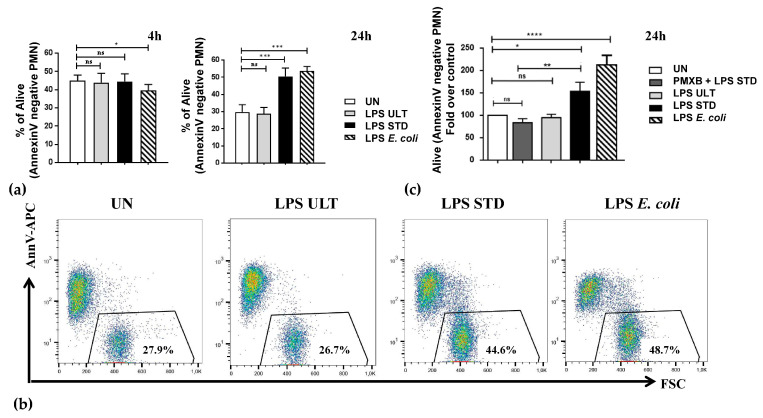
*P. gingivalis* LPS Standard inhibits neutrophil apoptosis. Wild-type HoxB8 neutrophils were either left untreated (UN) or stimulated for 4 h or 24 h with 1 μg/mL of *Pg*-derived LPS Ultrapure (“LPS *Pg* ULT” activates only Toll-like receptor 4 (TLR4)) or Standard (“LPS *Pg* STD” activates Toll-like receptor 2 (TLR2) and TLR4); *E. coli*-derived LPS served as a positive control. After indicated time-points, supernatants were collected and cells were harvested by 5-min treatment with accutase. (**a**) Cell survival was analyzed by flow cytometry by AnnexinV-APC staining. Quantification of results from three independent wild-type cell lines; bars show means +S.E.M. ANOVA followed by Bonferroni post-hoc test; ns-not significant, * *p* ≤ 0.05, *** *p* ≤ 0.001. (**b**) Representative dot plots showing Annexin V staining analyzed by FACS and FlowJo software. The percentage of Annexin V-negative cells is gated. (**c**) Cells were treated as in (**a**), but polymyxin B ("PMXB") at the final concentration of 100 μg/mL was added, where indicated, 30 min prior to LPS treatments. Quantification of results from three independent wild-type cell lines; bars show means and are presented as % of control +SEM. ANOVA followed by Bonferroni post-hoc test; * *p* ≤ 0.05, ** *p* ≤ 0.01, *** *p* ≤ 0.001, **** *p* ≤ 0.0001 ns-not significant.

Firstly, in order to identify the most optimal conditions for neutrophil activation by *P. gingivalis*-derived LPSs, dose and time course experiments using wild-type HoxB8 PMNs were performed. Three independent wild-type HoxB8 progenitor lines were differentiated for 4 days into neutrophils, which were subsequently treated for 4 or 24 h with graded doses of LPS Standard or LPS Ultrapure (0.01, 0.1, 1.0 and 10 μg/mL) isolated from *P. gingivalis*. *E. coli*-derived LPSs, binding only to TLR4 [11], served as a positive control. The flow cytometry analysis revealed that two purification variants of *P. gingivalis* endotoxin differentially influenced neutrophil survival and inflammatory functions. Standard *Pg* LPS significantly protected PMNs from apoptosis after 24 h (Figure 2a,b). This effect was dose-dependent (at concentrations of 0.1, 1.0 and 10 μg/mL; Supplementary File, Supplementary Figure S1a). In contrast, incubation with Ultrapure *Pg* LPS did not rescue neutrophils from apoptosis, even at the highest dose of 10 μg/mL (Supplementary Figure S1a), which indicated that signaling through TLR2 is essential in triggering pro-survival signaling in PMNs by *P. gingivalis*-derived endotoxins (Figure 2a,b). 

**Figure 3:**
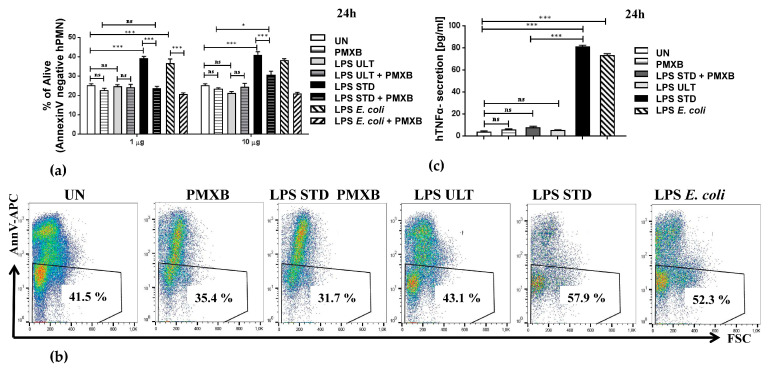
Primary human neutrophils respond similarly to *Pg* LPS as HoxB8 neutrophils. After primary human neutrophils isolation from density gradient, cells were treated with *P. gingivalis*-derived LPS (LPS ULT or LPS STD) or *E. coli*-derived endotoxin at indicated concentrations (1 μg/mL or 10 μg/mL), which served as a positive control. Where indicated polymyxin B at the final concentration of 100 μg/mL was added 30 min before LPS treatment. After 24 h-treatment, supernatants were collected and cells were harvested. (**a**) Cell survival was analyzed by flow cytometry by AnnexinV-APC staining. Presented representative results obtained from one out of four donors; bars show means +S.E.M. ANOVA followed by Bonferroni post-hoc test; ns-not significant, * *p* ≤ 0.05, ** *p* ≤ 0.01, *** *p* ≤ 0.001; (**b**) Representative dot plots showing Annexin V staining analyzed by FACS and FlowJo software. The percentage of Annexin V-negative cells is gated. (**c**) Cells were treated as in (**a**) and supernatants were collected at the indicated time-points. Production of hTNF-α was analyzed in supernatants by ELISA. Presented representative results obtained from one out of four donors. Bars show means +S.E.M. ANOVA followed by Bonferroni post-hoc test; *** *p* ≤ 0.001, ns-not significant.

Remarkably, prolonged survival in the presence of Standard *Pg* LPS was also noted in primary human neutrophils (Figure 3a,b and Supplementary Figure S2a). Importantly, this anti-apoptotic effect triggered by Standard *Pg* LPS could be neutralized by a 30-min pre-incubation with polymyxin B in murine (Figure 2c) as well as human neutrophils (Supplementary File, Supplementary Figure S2a).

### 2.3. Neutrophil Inflammatory Functions are Differentially Affected by Two Purification Variants of P. gingivalis Endotoxin

**Figure 4:**
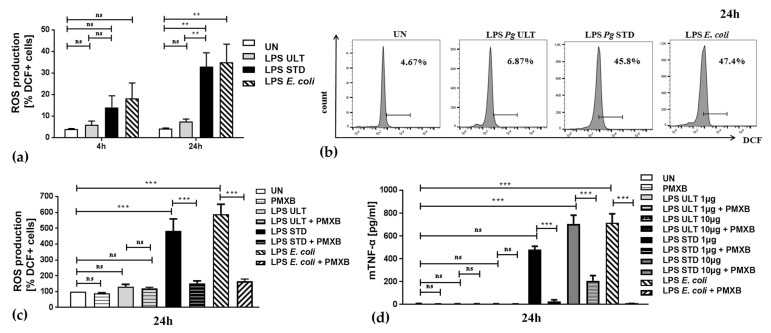
*P. gingivalis* LPS Standard enhances pro-inflammatory responses of murine neutrophils. Wild-type HoxB8 neutrophils were either left untreated (UN) or stimulated for 4 h or 24 h with 1 μg/mL of *Pg*-derived LPS Ultrapure or Standard, or *E. coli*-derived LPS. After indicated time-points, supernatants were collected and cells were harvested by 5-min treatment with accutase. (**a**) Harvested PMNs were stained with DCFH (dichlorofluorescein) for 30 min at 37 °C in the darkness and fluorescence of internalized oxidized DCF was analyzed by flow cytometry. Quantification of results from three independent wild-type cell lines. Bars show means +S.E.M. ANOVA followed by Bonferroni post-hoc test; ns-not significant, ** *p* ≤ 0.01. (**b**) representative histogram overlays showing internalized and oxidized fluorescent DCFH in comparison to the untreated control analyzed by flow cytometry and FlowJo software. The percentage of fluorescent cells is indicated. (**c**) Neutrophils were treated as in (**a**), but polymyxin B (PMXB) at the final concentration of 100 μg/mL was added (where indicated) 30 min prior to LPS treatments. Quantification of results from three independent wild-type cell lines; bars show means and are presented as % of control +SEM. ANOVA followed by Bonferroni post-hoc test; ns-not significant, ** *p* ≤ 0.01, *** *p* ≤ 0.001. (**d**) Neutrophils were treated as in (**a**) with indicated LPS concentrations (1 μg/mL or 10 μg/mL). Production of mTNF-α was analyzed in supernatants by ELISA. Quantification of results from six independent wild-type cell lines. Bars show means +S.E.M. ANOVA followed by Bonferroni post-hoc test; *** *p* ≤ 0.001, ns-not significant.

Recently, using the HoxB8 system as well as in vivo in experimental pneumococcal meningitis, we and others demonstrated that, in the presence of *E. coli*-derived LPSs or in the context of *E. coli* or *Streptococcus pneumoniae* infection, the survival of PMNs was extended [12,13]. Importantly, prolonged survival was accompanied by an exaggerated inflammatory response, which contributed to the observed symptoms of inflammatory disease [12,13,14]. Therefore, we then analyzed and compared PMN response to stimulation by different purification variants of LPSs. We discovered that neutrophils challenged for 24 h with 1 μg/mL Standard *Pg* endotoxin produced elevated ROS levels (Figure 4a,b). Concomitantly, the production of mTNF-α was also significantly enhanced in the presence of this TLR2- and TLR4-activating purification variant of *P. gingivalis* LPS (Figure 4d). Interestingly, Ultrapure *Pg* LPS, binding only to TLR4, did not show any pro-inflammatory effects (Figure 4a–d) even up to the 10 μg/mL concentration (Supplementary Figure S1b). In line with previously published data, stimulation with *E. coli* LPS induced the oxidative burst and mTNF-α secretion [13]. Notably, the production of this pro-inflammatory cytokine by neutrophils was almost two times higher in the presence of *E. coli*-derived LPS than Standard *Pg* LPS at a concentration of 1 μg/mL (479.8 pg/mL vs. 714.0 pg/mL; Figure 4d). Notably, at the concentration of 10 μg/mL of LPS STD the secretion of mTNFα by neutrophils (707.5 pg/mL) almost reached the levels of mTNFα production triggered by *E. coli* LPS 1 μg/mL. *Pg*-derived LPS Ultrapure (“LPS *Pg* ULT”) did not induce the cytokine secretion inflammatory response and even at the highest tested dose, i.e., 10 μg/mL, was very similar to the mTNFα levels generated by untreated controls (7.42 pg/mL vs. 7.72 pg/mL; Figure 4d). Remarkably, the application of polymyxin B not only abrogated the anti-apoptotic effect of *P. gingivalis*-derived LPS on PMNs (Figure 2c and Figure 3a,c), but also abolished neutrophil pro-inflammatory responses, since mTNF-α and reactive oxygen species levels were significantly reduced in the presence of polymyxin B (Figure 4c,d and Supplementary Figure S1b). Notably, polymyxin B also reduced mTNF-α production induced by *E. coli* LPS (Supplementary Figures S1b and S3). 

To exclude the possibility that the observed effects could be attributed to polymixin B off-target effects, we also tested a specific TLR4 inhibitor (CLI-095). Like the LPS inactivator (Figure 4d), CLI-095 significantly suppressed LPS Standard-mediated inflammatory responses (Supplementary File, Supplementary Figure S3).

These results strongly suggest that neutrophil activation by *P. gingivalis* endotoxin required concomitant activation of TLR2 and TLR4 in order to exert anti-apoptotic and pro-inflammatory effects. Importantly, the pro-inflammatory responses of primary human neutrophils, as measured by hTNF-α secretion, were also upregulated upon cell treatment with 1.0 μg/mL and 10 μg/mL *Pg* LPS Standard (Figure 3c and Supplementary Figure S2b). In contrast, Ultrapure endotoxin from *P. gingivalis* induced only a moderate effect at the highest used concentration, i.e., 10 μg/mL (Supplementary Figure S2b). Altogether, these data indicate that the survival and inflammatory responses of both murine HoxB8 neutrophils (Figure 4 and Supplementary Figure S1) and primary human neutrophils (Figure 3 and Supplementary Figure S2) were similarly manipulated by *P. gingivalis*-derived LPS Standard.

## 3. Discussion

Periodontal disease is arguably one of the most prevalent inflammatory conditions and affects about 20–50% of the population around the globe, thus representing a prominent public health burden [15,16]. Cigarette smoking, diabetes and osteoporosis were identified as the risk factors, yet infections with red complex bacteria, such as *P. gingivalis, Treponema denticola* and *Tannerella forsythia* [17] are regarded as fundamental for periodontitis development. The pathogenesis of this inflammatory disease is associated with an over-reactive host inflammatory response to periodontopathogens that accounts for the majority of periodontal tissue damage [18]. However, until now the impact of two purification variants of *P. gingivalis* lipopolysaccharide on neutrophil survival and pro-inflammatory functions has remained unraveled.

Here, we describe a new perspective to analyze neutrophil contribution in the progression of periodontitis, using the recently developed in vitro HoxB8 system [5]. This HoxB8 progenitor system is a very elegant in vitro model that enables the study of the survival and function of neutrophils [12,13]. Moreover, it is well suited not only for the analysis of PMN functions, but also for investigating apoptosis [5,6]. For cell death research, the HoxB8 system has the advantage of combining near-physiological conditions of apoptosis in non-transformed cells with the ease of the generation of large amounts of PMNs and the possibility of convenient genetic manipulation and biochemical analysis. Importantly, this system recapitulates the in vivo data [19,20], human data (Figure 3 and Supplementary Figure S2), and enables a detailed and precise analysis of PMN survival (Supplementary Figure S4) and functions.

Our experiments using *P. gingivalis*-derived Standard LPS, which activates both TLR4 and TLR2 signaling pathways, revealed that both murine (Figure 2 and Figure 4, and Supplementary Figure S1a) as well as human (Figure 3 and Supplementary Figure S2a) PMNs were strongly protected from spontaneous apoptosis in a dose-dependent manner (Supplementary Figures S1a and S2a). This endotoxin variant also triggered an inflammatory response, as measured by ROS production and the release of TNF-α. (Figure 4d, Supplementary Figures S1b and S2b). In contrast, Ultrapure *Pg* LPS, described to bind only to TLR4, neither exerted an anti-apoptotic activity, nor induced the activation of murine neutrophils (Figure 2 and Figure 4, Supplementary Figure S1), while on human cells Ultrapure endotoxin induced the effects only at the highest, already non-physiological concentration, i.e., 10 μg/mL (Supplementary Figure S2).

According to the manufacturer’s information, the Ultrapure LPS is obtained from regular LPS subjected to an additional enzymatic purification step to remove lipoproteins (https://www.invivogen.com/lps-pg). Consistently, already-published results using HEK293 cells overexpressing either TLR2 or TLR4 receptor confirmed that cell activation strongly depends on the purification variant of lipopolysaccharide [10]. Cells overexpressing human TLR2/CD14 were significantly activated only by Standard *Pg* LPS, while genetically modified TLR4/MD2/CD14 cells responded to both *Pg* endotoxin variants [10].

Furthermore, Nativel et al. [10] also analyzed the impact of graded doses of these two *P. gingivalis* endotoxin purification variants using murine macrophage RAW264.7 and J774.1 cell lines. In line with our observations, the authors also noted the inflammatory response only upon Standard *Pg* LPS stimulations, but not Ultrapure *Pg* LPS treatments, indicating exclusive TLR4-dependent macrophage activation. Future studies should verify whether the interaction between phagocytes and lipopolysaccharide may be further modulated by the conditions and possibly also depending on the stage of the infection with this periodontal pathogen.

In line with previous observations in vivo [21], we confirmed the importance of the cross talk between Toll-like receptors in the development of periodontal inflammation, since mice deficient in TLR2 or TLR4 did not suffer from alveolar bone resorption and an inflammatory reaction after polymicrobial infection with periodontal pathogens [21]. Notably, the observed TLR2-dependent effects on neutrophil survival and their inflammatory responses triggered by *P. gingivalis* are most probably PI3K/PKB (phosphoinositide 3-kinase/protein kinase B, also called Akt kinase) dependent [18,22]. Previous publications described the manipulation of this signaling pathway by *P. gingivalis*, since the substitution of TLR2-PI3K instead of TLR2-MyD88 signaling triggered the host inflammatory response that promoted observed bone resorption in mice, but concomitantly inhibited the bactericidal activity [18,22].

In summary, the results presented here, together with some earlier studies on macrophages [10,23], provide strong evidence that two purification variants of lipopolysaccharides activating different Toll-like receptors exert opposing actions on distinct immune cell populations. Moreover, our observations support already-published results and further indicate that *P. gingivalis* manipulates intracellular signaling pathways in order to inhibit neutrophil survival, induce the secretion of pro-inflammatory cytokines and trigger oxidative burst, all of which promote periodontal disease progression. Taken together, this might contribute to the observed tissue damage in an in vivo mouse model of periodontitis as well as in periodontitis patients [24,25]. It is especially important as this periodontopathogen is an asaccharolytic organism that requires peptides and hemin for growth [26], which are products of the inflammatory breakdown of the host connective tissue [27,28]. Therefore, the prolonged survival and hyperactivation of inflammatory responses provide *P. gingivalis* and the entire microbial community with access to essential nutrient resources.

## 4. Materials and Methods

### 4.1. HoxB8 Cell Lines and Cell Cultures

HoxB8 neutrophil progenitor cell lines from the bone marrow of C57BL/6 wild-type mice (kindly provided by Andreas Villunger, Medical University of Innsbruck, Austria) were established by retroviral transduction of HoxB8 and selection in the presence of SCF and β-estradiol as described before [5]. The estrogen-regulated HoxB8 expression plasmid was a kind gift of Hans Häcker (St. Jude Children’s Research Hospital, Memphis, TN, USA, MTA signed on 28.01.2018). In short, progenitors were cultured in Opti-MEM medium (Life Technologies, Warsaw, Poland), supplemented with 10% FCS (Sigma-Aldrich, Darmstadt, Germany), 250 μM L-glutamine, 100 U/mL penicillin and 100 μg/mL streptomycin, 30 mM β-mercaptoethanol, 1 μM β-estradiol (all from Sigma-Aldrich) and 1% supernatant from SCF-producing CHO cells (kindly provided by Hans Häcker). Differentiation in a medium containing 1% SCF supernatant was induced by the removal of estrogen. On day 4, cells were harvested, washed in PBS (Sigma-Aldrich), seeded at a density of 10^6^ cells/mL and stimulated. After stimulations, supernatants were collected and cells were harvested by 5-min treatment with accutase (Sigma-Aldrich), as activated neutrophils attach to the cell culture plastic surface (TPP, Trasadingen, Switzerland).

### 4.2. Murine and Human Neutrophil Isolations 

Primary mouse neutrophils were sorted from bone marrow of C57BL/6 mice by positive selection using the Miltenyi MACS purification system (Figure 1b) (Miltenyi Biotec, Teferow, Germany). After the harvest of bone marrow cells and red blood cell lysis, neutrophils were labeled with FITC-coupled anti-Gr-1 antibody followed by labeling with anti-FITC antibody-conjugated magnetic beads and passaged over MACS columns (Miltenyi Biotec). 

Primary human neutrophils (Figure 1b) were obtained from healthy volunteers. Neutrophils were isolated from peripheral EDTA blood by discontinuous density gradient separation consisting of lymphocyte separation medium 1077 and Histopaque 1119 (PAA, Pasching, Austria), followed by a discontinuous Percoll gradient separation (85/80/70/65%) (Sigma-Aldrich) as previously described [29]. Murine Gr-1-positive cells or human density gradient-purified cells were subjected to Giemsa staining (Sigma-Aldrich) immediately after isolation (Figure 1b).

Alternatively, neutrophils (Figure 3) were isolated as described before from granulocyte-enriched fractions, applied to centrifugation over a density gradient using a lymphocyte separation medium [30]. Neutrophils and erythrocytes were harvested as the high-density fraction and separated after 30 min of incubation with 1% polyvinyl alcohol (POCH, Warsaw, Poland). Neutrophils were collected from the upper layer and after centrifugation (280 ×g, 10 min), the residual erythrocytes were removed by lysis in water. Neutrophils were resuspended in DMEM without phenol red (Life Technologies) containing 5% serum. Immediately after isolation, cells were seeded at a density of 10^6^ cells/mL and stimulated. After stimulations, supernatants were collected and cells were harvested and analyzed by flow cytometry. Peripheral blood from de-identified human donors was obtained from the Red Cross (Krakow, Poland).

### 4.3. Immunofluorescence and Microscopic Analysis

The percentage of viable cells was determined using Annexin-V-APC (BioLegend, San Diego, CA, USA), while expression of cell surface markers was measured by staining cells with anti-c-kit-PE-Cy7, anti-Gr-1-PE and anti-Mac-1-APC (all from BioLegend) according to the manufacturer’s protocol followed by flow cytometry analysis on a FACS Calibur (Becton Dickinson, San Diego, CA, USA). FACS data were analyzed with FlowJo Software (Version X for Windows, FloJo LLC, Ashland, OR, USA).

Monitoring of cellular morphology (Figure 1b) was performed on cytospins from cultures of progenitors or day 4 differentiated HoxB8 cells as well as from freshly isolated primary mouse or human neutrophils. Cytospin slides were incubated with Giemsa (Sigma-Aldrich) solution after methanol fixation (Sigma-Aldrich). Analysis by brightfield microscopy was performed using a Keyence BZ9000 microscope at a magnification of 40× (Keyence, Itasca, IL, USA).

### 4.4. Neutrophil Stimulations

Cells were stimulated with LPS Standard or LPS Ultrapure from *P. gingivalis* (Invivogen, San Diego, CA, USA; cat. nos. tlrlpglp and tlrlppglps), or LPS from *E. coli* O55:B5 (Sigma-Aldrich, cat. no. L2637), which served as a positive control. Where indicated, cells were pre-treated with LPS inactivator polymyxin B (100 μg/mL, Invivogen, cat no. tlrlpmb) for 30 min prior to LPS addition, as published before [31].

### 4.5. Functional Analysis

Reactive oxygen species production was analyzed as described previously [14]. In short, after indicated time points, cells were harvested and 10^5^ cells were stained for 30 min at 37 °C with 20 μM 2′,7′-dichlorofluorescin diacetate (Sigma-Aldrich) and washed extensively with ice-cold PBS (Sigma-Aldrich); fluorescence was analyzed by flow cytometry on a FACS Calibur (BD). Levels of mTNF-α or hTNF-α were measured by ELISA in collected supernatants according to the manufacturer’s protocols (RnD Systems, Wiesbaden, Germany and Life Technologies respectively) and as published before [10,14]. Absorbance was measured using Flex Station 3 (Molecular Devices, San Hose, CA, USA) or Infinite M200 Plate Reader (Tecan, Maennedorf, Switzerland).

### 4.6. Ethics Statement 

In relation to Figure 1b, Blood was collected from healthy volunteers who provided oral informed consent for the collection of samples and subsequent cell isolation and analysis. For human subject confidentiality assurances, blood material was de-identified; thus, this manuscript adheres to appropriate exclusions from human subject approval in accordance with local and national legislation and the Declaration of Helsinki. The studies involving animals (neutrophil isolation from murine bone marrow) were performed according to the national Protection of Animals Act and were approved by the Regierungspraesidium Freiburg, Freiburg, Germany (approval code: X-18/03K).

In relation to Figure 3, Human blood for PMN isolation was purchased from Red Cross, Krakow, Poland as described before [30]. The Red Cross de-identified blood materials as appropriate for the confidentiality assurance of human subjects. Thus, this study adheres to appropriate exclusions from the approval of human subjects.

In relation to Figure 2 and Figure 4, HoxB8 progenitor lines were kindly given by Professor Andreas Villunger and were generated as described before [32].

### 4.7. Statistical Analysis

Data are presented as the mean +SEM or as a percentage of control +SEM. Statistical analysis was performed using ANOVA followed by a Bonferroni post-hoc test, applying the PRISM Graphpad software (version 7 for Windows, PraphPad Software, San Diego, CA, USA). *p*-values < 0.05 were considered to indicate statistically significant differences.

## 5. Conclusions

The differences between mouse and human immunology should be taken into account when using mice as an experimental tool, especially during the very late phases of preclinical drug development. The ratio between neutrophils and lymphocytes is quite different, as neutrophils are the most abundant white blood cells present in the human body, while in mice only up to 25% of white blood cells are neutrophils [33]. However, in many cases, mice perfectly mirror human biology [34], including periodontitis. In both species, neutrophils represent the overwhelming majority of the leukocytes recruited to the gingival crevice in PD patients as well as in chamber models of experimental periodontitis in mice [21,35]. 

HoxB8 progenitor lines can be easily genetically modified in vitro (knockout, knockdown or overexpression) in order to achieve desirable results, and in support of the EU-3R directive, this reduces the extensive use of laboratory animals. Therefore, this HoxB8 model system is an ideal tool to study the survival and inflammatory functions of neutrophils as well as the molecular mechanisms of their contribution to inflammatory diseases, including periodontal disease. Using this model, our findings revealed that challenge with *P. gingivalis*-derived LPS Standard, which utilizes TLR2 and TLR4, prolonged both human and murine neutrophil survival as well as increased the production of reactive oxygen species and TNF-α. This can certainly contribute to detrimental tissue destruction, leading to the development and progression of periodontitis. Importantly, these negative outcomes could be mitigated by the inhibition of this virulence factor by polymyxin B. Notably, the effects of polymyxin B or polymyxin E (colistin) therapy are currently being evaluated in clinical trials in patients with multi-drug resistant pathogens, septic shock or acute kidney injury [36]. In May 2019, a clinical trial devoted to determining the roles of TLR2 and TLR4 in periodontal disease (https://clinicaltrials.gov/ct2/show/NCT04201912) started. However, no clinical trials testing the possibility to treat periodontitis patients with polymyxin B or E have been reported so far. Polymyxins were developed in the 1940s and the intravenous application of these polypeptide antibiotics exert high toxicity [37]. It is likely, however, that topical oral application of these compounds for the treatment of periodontitis patients might have only minimal or negligible undesired effects. Therefore, the application of LPS inhibitors might be a novel therapy for periodontitis, targeting aberrant neutrophil survival and functions. Future studies should be initiated to expand our understanding of neutrophil manipulation by periodontopathogens to better define new therapeutic strategies.

## Supplementary Materials

The following are available online at https://www.mdpi.com/2076-0817/9/7/530/s1, Supplementary File: Supplementary Methods–Cell stimulations; Figure S1: Murine neutrophils response to graded doses of *Pg* LPS; Figure S2: Human neutrophils respond to *Pg* LPS in a dose-dependent manner; Figure S3: Toll-like receptors 2 and 4 cross-talk is essential for neutrophil response to *Pg* LPS; Figure S4: Staurosporine treatment as a positive control for apoptosis induction and verification of the gating strategy.

## Abbreviations

SCF—Stem Cell Factor; *Pg*—*Porphyromonas gingivalis*; PMN—polymorphonuclear leukocyte; HoxB8—homeobox B8, TLR—Toll-like receptor; TNF-α—Tumor Necrosis Factor-alpha, LPS—lipopolisaccharide; ROS—reactive oxygen species; GM-CSF—granulocyte-macrophage colony-stimulating factor; CHO—chinese hamster ovary cells; *E. coli*—*Escherichia coli*; BM—bone marrow; PB—peripheral blood.
