# Supplementary material for: Application of the In Vitro HoxB8 Model System to Characterize the Contributions of Neutrophil–LPS Interaction to Periodontal Disease
Source: Pathogens. 2020 Jul 1;9(7):530. doi: 10.3390/pathogens9070530 (PMC7399906; doi:10.3390/pathogens9070530)
Supplement: Supplementary file 1 [file pathogens-09-00530-s001.pdf]

Supplementary Material

Supplementary File

Supplementary Methods–Cell stimulations

Cells were stimulated with LPS, which served as a positive control. Where indicated, cells were pre-treated with LPS inactivator polymyxin B at 100 µg/mL final concentration (Invivogen) or CLI-095 1 µM final concentration (Invivogen, nr kat. tlrl-cli95).

Alternatively, apoptosis was induced by 1 µM staurosporine (Sigma-Aldrich), which served as a positive control and verified our gating strategy (Supplementary Figure S4).

Supplementary Figures

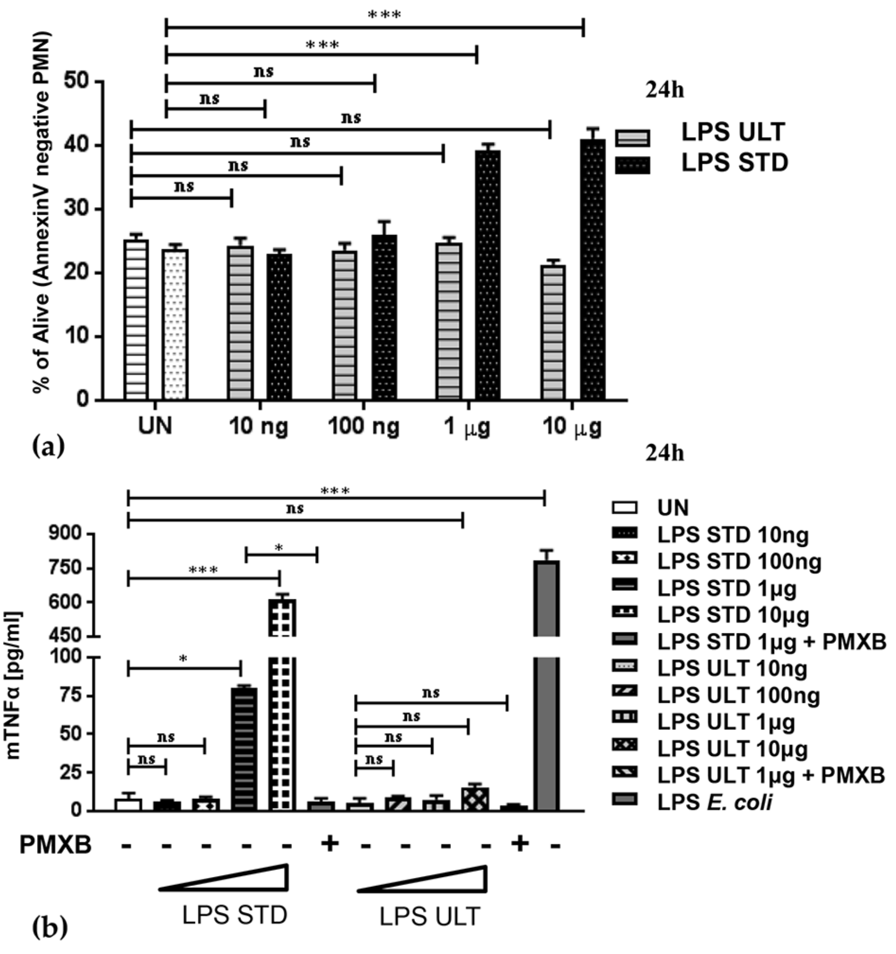

Figure S1. Murine neutrophils response to graded doses of *Pg* LPS. Murine HoxB8 neutrophils were stimulated for 24 h with graded doses of *P. gingivalis*-derived LPS: Ultra-pure or Standard i.e. 10 ng/mL, 100 ng/mL, 1.0 µg/mL or 10 µg/mL. After treatment supernatants were collected, while activated cells were detached by 5-minute treatment with accutase. (**a**) Cell survival was analyzed by AnnexinV and flow cytometric analysis. Quantification of results from 3 independent experiments; Bars show means +S.E.M. ANOVA followed by Bonferroni post-hoc test; ns- not significant, *** $p \leq 0.001$. (**b**) Neutrophils were treated as in (**a**), but polymyxin B (PMXB) at the final concentration of 100 µg/mL was added (where indicated) 30 minutes prior to LPS treatments. Production of mTNF-α was analyzed in supernatants by ELISA. Quantification of results from 3 independent experiments. Bars show means +S.E.M. ANOVA followed by Bonferroni post-hoc test; * $p \leq 0.05$,*** $p \leq 0.001$, ns-not significant.

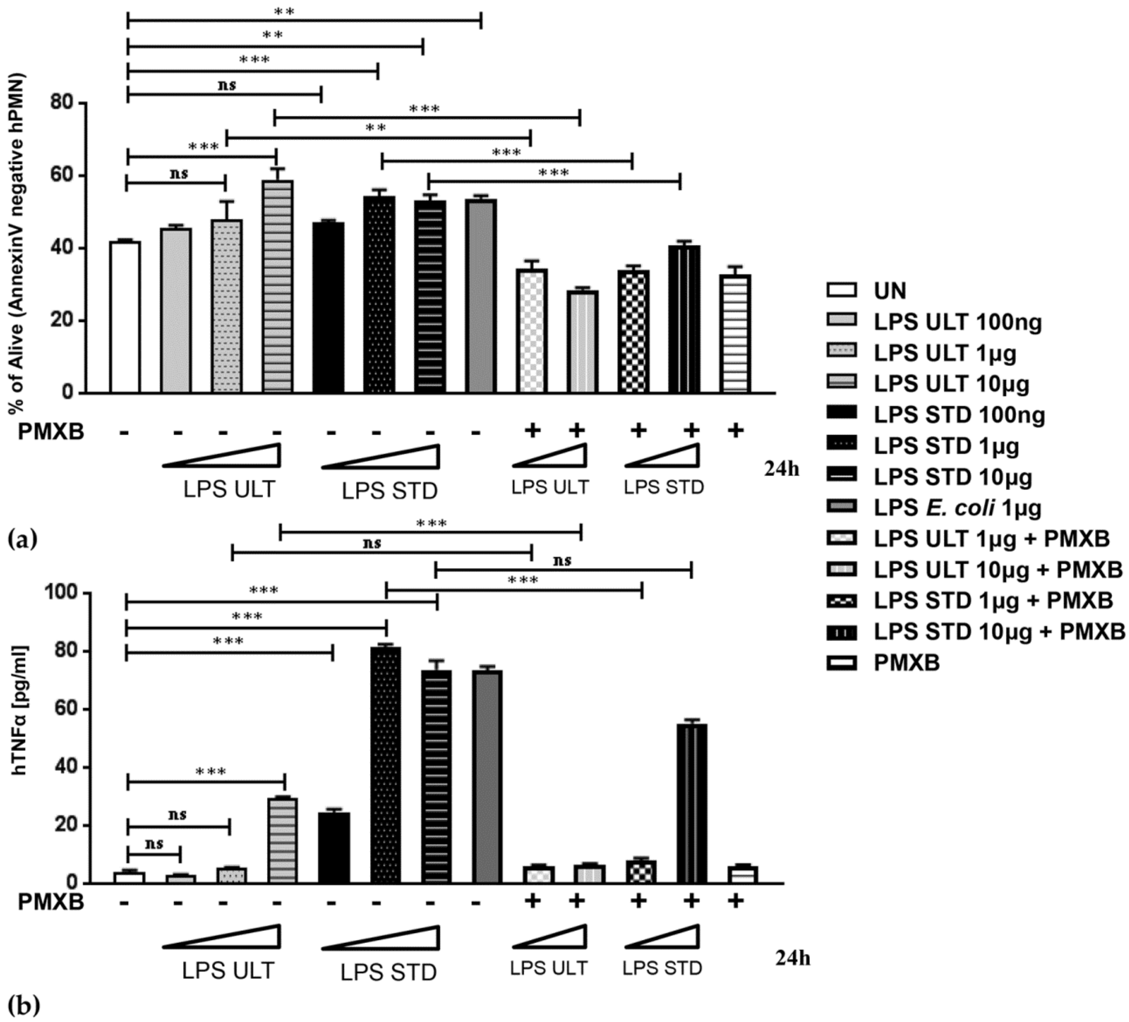

Figure S2. Human neutrophils respond to *Pg* LPS in a dose-dependent manner. After isolation primary human neutrophils were treated with increasing doses of *P. gingivalis*-isolated LPS ULT or STD (100ng/mL, 1.0 µg/mL, or 10 µg/mL.) 1.0 µg/mL *E. coli*-derived endotoxin was used as a positive control. Where indicated polymyxin B at the final concentration of 100 µg/mL was added 30 minutes before LPS treatment. After 24 hour-treatment, supernatants were collected and cells were harvested. (**a**) Cell survival was analyzed by flow cytometry by AnnexinV-APC staining. Presented representative results obtained from one out of four donors; Bars show means +S.E.M. ANOVA followed by Bonferroni post-hoc test; ** $p \leq 0.01$, *** $p \leq 0.001$, ns- not significant; (**b**) Production of hTNF-α was analyzed in supernatants by ELISA. Presented representative results obtained from one out of four donors. Bars show means +S.E.M. ANOVA followed by Bonferroni post-hoc test; *** $p \leq 0.001$, ns- not significant.

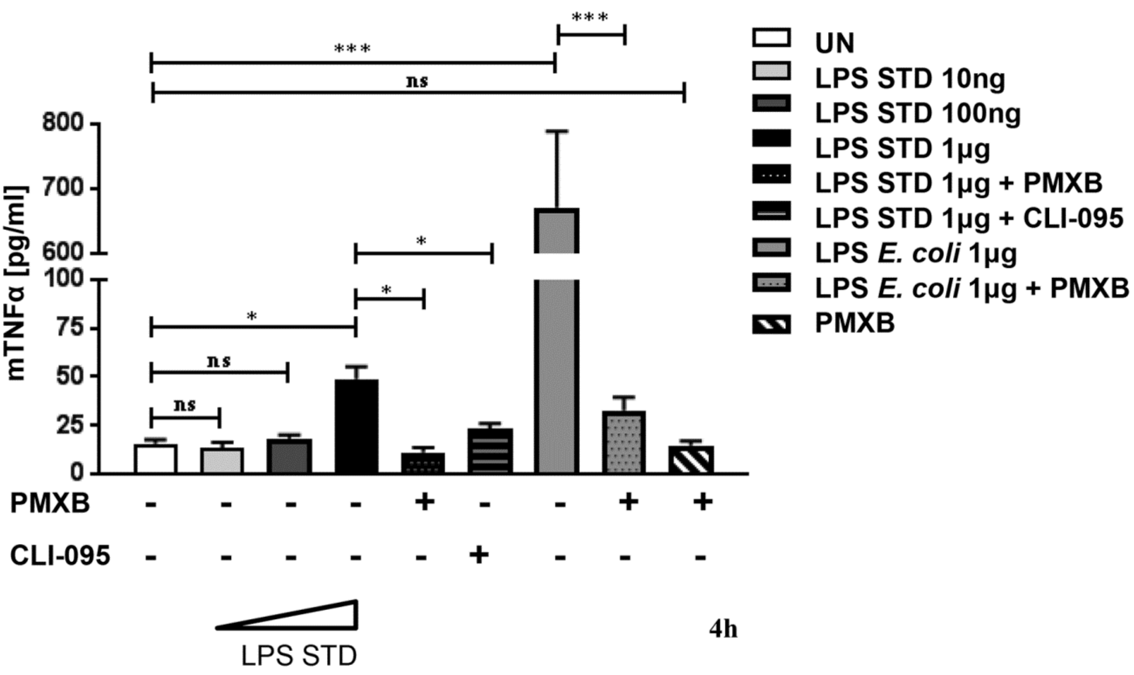

Figure S3. Toll-like receptors 2 and 4 cross-talk is essential for neutrophil response to *Pg* LPS. HoxB8 wild-type neutrophils were subjected to 4 h-treatment with graded doses of *P. gingivalis*-derived LPS Standard i.e. 10 ng/mL, 100 ng/mL, 1.0 µg/mL or 10 µg/mL. Where indicated polymyxin B (PMXB) or TLR4 inhibitor (CLI-095) at the final concentrations of 100 µg/mL or 1 µM/mL respectively were added 30 minutes prior to LPS treatments. Cell supernatants were collected after incubation, while remaining cells were detached by 5-minute treatment with accutase. (**a**) Production of mTNF-α was analyzed in supernatants by ELISA. Quantification of results from 3 independent experiments. Bars show means +S.E.M. ANOVA followed by Bonferroni post-hoc test; * $p \leq 0.05$, *** $p \leq 0.001$, ns-not significant.

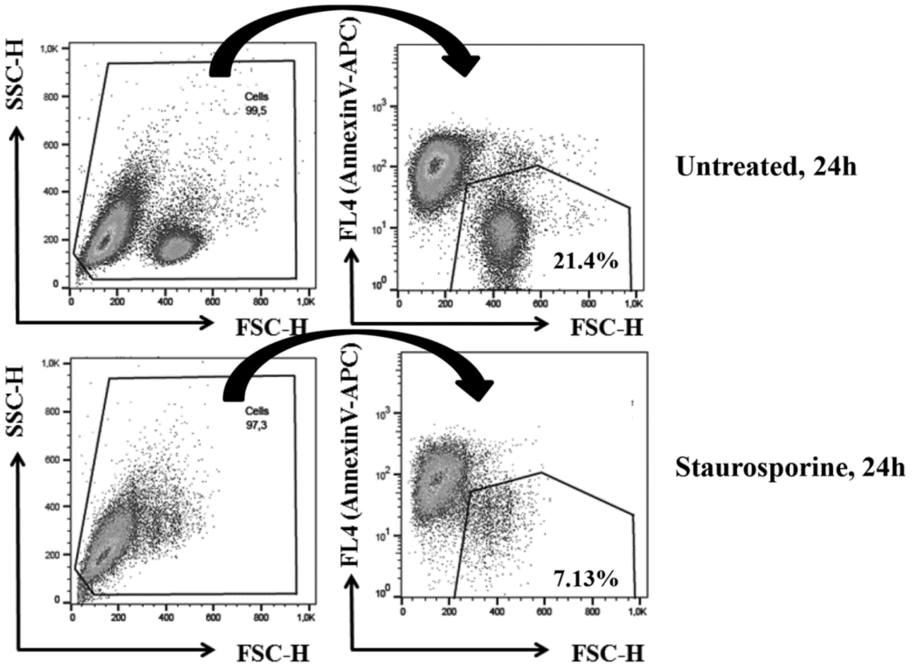

Figure S4. Staurosporine treatment as a positive control for apoptosis induction and verification of the gating strategy. HoxB8 wild-type neutrophils were subjected to 24 h-treatment with 1 µM staurosporine. Cells were harvested by 5-minute treatment with accutase. Cell survival was analyzed

by flow cytometry by AnnexinV-APC staining. In order to exclude cell debris from analysis, we excluded very small events visible on the dot plots in the left panels (SSC vs FSC) and called the remaining population "Cells". This population was next subgated and analyzed according to the Annexin V positivity (Y-axis, right panel).
